# Overemployment and underemployment of part-time pharmacists: Prevalence and connection to work-life characteristics

**DOI:** 10.1016/j.rcsop.2025.100673

**Published:** 2025-10-24

**Authors:** David A. Mott, William R. Doucette, Eilan Alhersh, Vibhuti Arya, Brianne K. Bakken, Caroline Gaither, David H. Kreling, Jon C. Schommer, Matthew Witry

**Affiliations:** a School of Pharmacy, University of Wisconsin-Madison 777, Highland Ave. Madison, WI 53705, USA; b College of Pharmacy, University of Iowa, Iowa City, IA 52242, USA; c College of Pharmacy and Health Sciences, St. John's University, Queens, NY 11439, USA; d The University of Iowa College of Nursing, Iowa City, IA 52242, USA; e University of Minnesota College of Pharmacy, Minneapolis, MN 55455, USA

**Keywords:** Pharmacist, Underemployment, Part-time, Work-life, Work-schedule control

## Abstract

**Background:**

Overemployment and underemployment are associated with fluctuations in labor supply and can negatively impact psychosocial aspects of work and worker health.

**Objectives:**

The objectives of this study were to 1) determine the prevalence and characteristics of underemployment, overemployment and matched employment among part-time pharmacists; 2) examine differences in actual and ideal hours worked for overemployed and underemployed part-time pharmacists; and 3) test associations between overemployment, underemployment and matched employment and part-time pharmacists' perceptions of job quality and work-life characteristics.

**Methods:**

Data for 636 pharmacists self-reporting working part-time (≤ 30 h/week) were extracted from the 2019 National Pharmacists Workforce Study. The difference in self-reported actual and ideal hours worked weekly was calculated and used to classify part-time pharmacists as overemployed, underemployed or matched employed. Differences between the variables were tested with multivariate ordinary least squares regression models.

**Results:**

Being matched employed was most common (41.3 %) followed by underemployed (34.9 %) and being overemployed (23.8 %). Of underemployed respondents, over half (54.1 %) reported wanting to work full-time, which likely is reflective of the relatively loose national pharmacist labor market in 2019. Overemployed and underemployed respondents reported significantly lower levels of several of the work-life characteristics relative to those with matched employment.

**Conclusion:**

The higher rate of underemployment among pharmacists working part-time is consistent with the surplus of US pharmacists in 2019. The results show that for pharmacists working part-time, a lack of control over how much they work is negatively associated with job quality and work-life characteristics relative to pharmacists with work schedule control.

## Highlights

• This analysis is among the first to examine overemployment, underemployment, and matched employment among part-time pharmacists.
• Underemployment was more common than overemployment, with 54.1 % of underemployed pharmacists expressing a desire for full-time work.
• Over- and underemployment were negatively associated with work-life wellbeing compared to matched employment.
• Future research could explore the causes and consequences of limited work schedule control among pharmacists.

## Introduction

In the United States, between 2000 and 2021, the number of pharmacy professional degree (i.e., Doctor of Pharmacy (PharmD)) graduates from schools and colleges of pharmacy increased from 7000 to 14,223, peaking at 14,905 in 2018, leading to reports that the shortage of pharmacists subsided.^1^, ^2^, ^3^, ^4^ Additional evidence of pharmacist labor market loosening (i.e., a surplus of pharmacists) was the decrease in annual nominal mean hourly wage growth between May 2010 and May 2019 and the relatively flat annual nominal mean hourly wage growth between May 2019 and May 2021.5., 6., 7., 8., 9. Despite the increase in graduates, 11.5 % of actively practicing pharmacists in 2019 reported a shortage of pharmacists in their local practice area.^10^^,^^11^ Government projections report a current and future pharmacist shortage in the US.^12^

Actively practicing pharmacists working part-time (i.e., working 30 or fewer hours per week) has been a notable feature of the pharmacist labor market for many years.^13^, ^14^, ^15^, ^16^, ^17^, ^18^, ^19^ Several characteristics of the pharmacist labor market contribute to pharmacists working part-time, including a shortage of pharmacists, similar wage rates for full-time and part-time work, an increasing proportion of women practicing pharmacy, and an increasing proportion of male pharmacists at or nearing retirement-age.^13^^,^^14^^,^^18^^,^^19^ Past research examined trends in part-time work among pharmacists, gender and age differences in part-time work, and reasons pharmacists worked part-time.^13^^,^^14^^,^^18^^,^^19^ Results from the 2019 National Pharmacist Workforce Study (NPWS) showed that the proportion of actively practicing pharmacists working part-time was 14.6 %, a decrease from 23.7 % in 2009 and 17.7 % in 2014.^10^^,^^20^^,^^21^ The decreasing trend is consistent with evidence suggesting the pharmacist labor market was loosening.

Another important characteristic of part-time work is the differential between the ideal number of hours a person would like to work and the actual number of hours that they do work.^22^ Working less than the number of hours a worker would like to work (i.e., underemployment) and working more hours that a worker would like to work (i.e., overemployment) are important aspects of working part-time since work is a significant part of workers' lives, influencing their financial situation, self-esteem, involvement in the community and their professional identity.^23^, ^24^, 25., 26, 27

Both underemployment and overemployment can have negative psychosocial effects on workers, potentially leading to poor mental and physical health.^28^^,^^29^ Underemployment and overemployment can lead to increased stress and poor experiences at work due to unstable and unpredictable weekly work hours resulting from poor control over work schedules.^30^ Unstable and unpredictable work hours can contribute to income instability, which may lead to housing and food insecurity and the reduced ability to cover medical costs and costs associated with health generating activities, such as eating healthy and exercising regularly.^31^^,^^32^ A lack of control over work schedules can lead to heightened stress due to the inability to plan for and control time spent in leisure and family activities.^33^

Excess labor supply can lead to increased underemployment which can come from overall economic downturn or industry specific changes.^34^^,^^35^ Such changes may include new job changes, technologies and increases in employers' nonwage costs, such as health insurance, that increase the cost of full-time work.^34^ The results can be limited work hours for employees and employers incentivized to hire more part-time workers relative to full-time workers.^36^, ^37^, ^38^ If labor shortages emerge, workers leave jobs in which they are underemployed.^39^ During periods of labor shortage, overemployment occurs when employers cannot find enough labor to work hours employers demand which, in turn, drives wages higher.^40^ Faced with higher wages, workers can choose to work fewer hours, substituting leisure time for income.^41^

The Bureau of Labor Statistics (BLS) measures the prevalence of underemployment or “working part-time due to economic reasons” as the proportion of all workers who are working less than full-time (i.e., part-time) but would like to be working full-time.^42^ Another measure of underemployment includes part-time workers who would like to work any number of additional hours per week, not just to a level of full-time employment.^22^ In 2016 and 2023, approximately 4 % of the US workforce was underemployed according to the BLS measure.^43^ Estimates using the broader measure of underemployment show that 8–11 % of US workers were underemployed in 2016.^29^ On the other end of the continuum is overemployment, or the proportion of workers who would like to work fewer hours than they currently work. There is no measure for overemployment of part-time workers by BLS.^42^

Although studies show that part-time work by pharmacists is associated with positive work life outcomes relative to pharmacists working full-time, no studies known to the authors have examined the prevalence of pharmacists working part-time who are underemployed, overemployed, or matched employed (i.e., working the number of hours they want to work).^44^ This is significant given the hypothesized positive relationship between a surplus of workers and the prevalence of underemployment.^34^, ^35^, ^36^, ^37^, ^38^ Additionally, although it is hypothesized that underemployment and overemployment are related to psychosocial aspects of work, no known study has examined relationships between underemployment and overemployment on work-life characteristics for pharmacists working part-time.28., 29, 30.^,^^33^ Filling the gaps in the pharmacy literature is important given the periodic fluctuations in the loosening and tightening of the pharmacist labor market and the corresponding hypothesized association with the prevalence of underemployment and overemployment, respectively, among pharmacists working part-time.^2^, ^3^, ^4^^,^^12^ Second, addressing the gaps in the literature is important given the need to improve work-life characteristics for pharmacists as they experience work life issues such as increased job stress, burnout, and reduced job satisfaction.^10^^,^^21^^,^^45^, ^46^, ^47^, ^48^

### Study objectives

Overall, the purpose of this study was to address gaps in the pharmacy literature related to characteristics and outcomes of part-time work among pharmacists. The objectives of this study were to 1) determine the prevalence and characteristics of underemployment, overemployment and matched employment among part-time pharmacists; 2) examine differences in actual and ideal hours worked for overemployed and underemployed part-time pharmacists; and 3) test associations between overemployment, underemployment and matched employment and part-time pharmacists' perceptions of job quality and work-life characteristics.

## Methods

### Data

The study uses unique data about hours worked, desired hours worked, and work life characteristics collected from pharmacists as part of the 2019 NPWS.^10^ Data were collected using electronic survey methods. A total of 94,803 emails containing the survey link were sent to sampled licensed pharmacists obtained from the National Association of Boards of Pharmacy Foundation. Across the three email contact waves, the average email open rate was 27.4 %, the average survey link click rate was 11.7 %, and the average undeliverable (i.e., bounce) rate was 1.4 %.^10^ Data were collected from May 22 to July 8, 2019. A description of the electronic survey methods and assessment of nonresponse bias and potential limitations are reported elsewhere.^49^

The survey questionnaire collected information from licensed pharmacists about their employment status, work setting, hours worked, work characteristics, work-life characteristics and demographics. A total of 4363 responding pharmacists reported actively practicing pharmacy (i.e., working as a pharmacist) and provided information to be considered a usable response (i.e., no missing data for age, gender, practice setting, and actual hours worked per week). Of the responding actively practicing pharmacists, a total of 636 respondents (14.6 %) were categized as working part-time based on reported actual hours worked per week (i.e., 30 or fewer hours per week) and were the sample for this study.^14^^,^^15^ This study was approved by the Institutional Review Board at an author's institution.

*Measures.*

### Underemployment, overemployment, and matched employment

The survey questionnaire allowed respondents to report the actual number of hours worked each week and the ideal number of hours they would choose to work each week. The weekly hours worked differential was defined as the difference between the ideal hours and the actual hours worked each week and was calculated for each respondent. Respondents working part-time for whom the weekly hours worked differential was positive (i.e., ideal hours > actual hours) were categorized as underemployed. Respondents for whom the weekly hours worked differential was negative (i.e., ideal hours < actual hours) were categorized as overemployed. Respondents were categorized as “matched employed” if the weekly hours worked differential was zero.

### Job quality

Respondents who reported that they worked part-time could respond to six items describing characteristics of their part-time job. The six items included the ability to pick the hours and days to work each week, whether hours worked were consistent across weeks, whether respondents were required to work at one location for their primary employer, whether the work schedule (i.e., hourly shifts) was favorable, and whether the work schedule was not provided on short notice.^50^ The rating scale is contained in Table 2. A summed score across the six items was calculated for each respondent. A higher score represented a more favorable or higher quality part-time job.

### Work-life characteristics

Work-life characteristics included job stress, job satisfaction, work-home conflict, professional fulfillment, work exhaustion and interpersonal disengagement. Job stress was measured using five items describing characteristics of respondents' work settings (i.e., staffing levels, workload, dealing with difficult patients, not having enough information about a patient's condition, and fearing a patient would be harmed by a medication error) that allowed respondents to report the level of stress associated with each item.^51^ Job satisfaction was measured with three items and work-home conflict was measured using a single item.^52^^,^^53^ A summed score for job stress and job satisfaction was calculated across all items in each scale.

The Professional Fulfillment Index (PFI) is a 16-item measure that assesses two aspects of wellbeing, professional fulfillment, and burnout and was developed initially to assess physicians.^54^ The PFI was used in previous studies of pharmacists.^55^ Professional fulfillment (PF) was measured using 6 items. Burnout consisted of two dimensions, work exhaustion (WE) (4 items) and interprofessional disengagement (ID) (6 items). The ID scale included three items each about relationships with colleagues and patients, respectively. Items related to relationships with patients were not included in the analysis since many respondents did not have interactions with patients and did not respond to the items about relationships with patients. Additionally, analysis of the means for items about relationships with colleagues and relationships with patients were not statistically significantly different. For each respondent, a PF, ID and WE score was the item mean across all items included to measure each construct. The rating scales used to evaluate each work-life characteristic are contained in Table 2.

### Work setting and demographic variables

For respondents who reported actively practicing pharmacy, an item allowed respondents to report their current practice setting from a list of 12 options. If a respondent reported practicing in a community practice setting, an additional item allowed the respondent to report the type of community practice setting (i.e., independent community pharmacy, small chain, large chain, mass merchandiser, supermarket). If a respondent reported working in a hospital/health system practice setting, an additional item allowed the respondent to report if they worked in a health system retail setting. A condensed work setting variable was created consisting of 5 categories: (1) independent, small chain, health system retail; (2) large chain, mass merchandiser, supermarket; (3) inpatient hospital; (4) ambulatory care; (5) other. Demographic variables included in this study were age, gender, race, and marital status.

### Data analysis

Descriptive statistics were calculated for all respondents who reported working part-time and those categorized as underemployed, overemployed and matched employed. Bivariate analyses testing the association between categories of part-time work and work setting and demographic variables were tested with the chi-squared test.

Mean differences in actual hours worked per week, ideal hours to work per week, the hours worked differential, job quality and each work-life characteristic across the categories of part-time work were tested with one-way ANOVA. Ordinary least squares regression models were estimated to examine the relationship between underemployment, overemployment and matched employment with job quality and each work-life characteristic controlling for work setting, demographic characteristics, and actual hours worked weekly. Differences between regression coefficients for matched employment and overemployment and underemployment were tested by excluding the matched employment group in the models. To test for differences between regression coefficients for overemployment and underemployment, planned comparisons were tested. Tests of assumptions made for the use of parametric statistics and ordinary least squares regression analysis were conducted. All statistical tests were evaluated using an a priori significance level of 0.05. All analyses were conducted using SPSS Statistics 25.

## Results

Of the 636 useable responses working part-time, a total of 11 respondents did not report their ideal weekly hours to work and could not be categorized as underemployed, matched employed or overemployed. Table 1 contains the characteristics of respondents actively practicing pharmacy, working part-time overall, and who were categorized as underemployed, overemployed and matched employed. Overall, approximately 20 % of respondents working part-time were age 65 years or older (17.9 %) and over 70 % were female (71.9 %). Among female respondents reporting working part-time, 15.2 % were 59 years or older compared to 63.6 % of male respondents reporting working part-time (data not shown).

**Table 1:** Characteristics of All Respondents, Respondents Reporting Working Part-Time and Classified as Underemployed, Matched Employed, and Overemployed, and Total Respondents Reporting Working Part-Time.

		Categories of Part-Time Work		
	Respondents Working Part-Time	Underemployed	Matched Employed	Overemployed
	N (Col %)	N (Row %)		
Total	625^1^	218 (34.9)	258 (41.3)	149 (23.8)
Characteristics				
Age*				
Up to 35	109 (17.4)	48 (44.0)	42 (38.5)	19 (17.4)
36–45	105 (16.8)	39 (37.1)	35 (33.3)	31 (29.5)
46–55	152 (24.3)	54 (35.5)	59 (38.8)	39 (25.7)
56–65	147 (23.5)	54 (36.7)	56 (38.1)	37 (25.2)
Over 65	112 (17.9)	23 (20.5)	66 (58.9)	23 (20.5)
Gender*				
Female	447 (71.5)	153 (34.2)	181 (40.5)	113 (25.3)
Male	176 (28.2)	65 (36.9)	75 (42.6)	36 (20.5)
Non-binary	2 (0.3)	0	2 (100.0)	0
**Ethnicity**				
White	501 (80.2)	169 (33.7)	215 (42.9)	117 (23.4)
Asian	60 (9.6)	24 (40.0)	26 (43.3)	10 (16.7)
Black	32 (5.1)	15 (46.9)	5 (15.6)	12 (37.5)
Other	28 (4.5)	9 (32.1)	10 (35.7)	9 (32.1)
Missing	4 (0.6)	1 (25.0)	2 (50.0)	1 (25.0)
Marital Status*				
Married	521 (83.4)	165 (31.7)	233 (44.7)	123 (23.6)
Divorced/Widowed/Separated	50 (8.0)	18 (36.0)	17 (34.0)	15 (30.0)
Single	51 (8.2)	34 (66.7)	8 (15.7)	9 (17.6)
Missing	3 (0.4)	1 (33.3)	0	2 (67.7)
Setting*				
Large Chain, Mass Merchandiser, Supermarket	185 (29.6)	75 (40.5)	65 (35.1)	45 (24.3)
Independent, Small Chain, Health system retail	146 (23.4)	56 (38.4)	62 (42.5)	28 (19.2)
Hospital inpatient	154 (24.6)	41 (26.6)	69 (44.8)	44 (28.6)
Other^2^	100 (16.0)	33 (33.0)	44 (44.0)	23 (23.0)
Outpatient/MD clinic	40 (6.4)	13 (32.5)	18 (45.0)	9 (22.5)

1 Total respondents working part-time is the sum of respondents across the categories of part-time work.

2 Other setting included academia, nursing home/LTC, home health/infusion, industry, mail order, managed care/PBM, specialty pharmacy, nuclear pharmacy, regulatory. * Chi-squared test of association between characteristic and category of part-time work, *p* < 0.05.

Being matched employed was most common (41.3 %) followed by underemployed (34.9 %) and being overemployed was least common (23.8 %). Being underemployed, overemployed or matched employed varied significantly related to age, marital status and practice setting. A significantly higher proportion of respondents 35 years or younger reported being underemployed (44.0 %) and a significantly higher proportion of respondents 65 years and older reported being matched employed (58.9 %). A significantly larger percentage of respondents working in community settings (i.e., large chain, mass merchandiser, supermarket (40.5 %) and independent, small chain, and health system retail practice settings (38.4 %)) were underemployed relative to the other practice settings. A significantly higher proportion of single respondents were underemployed.

Table 2 contains a summary of reported actual and ideal weekly work hours, perceptions of job quality and work-life characteristics for respondents overall and categorized as underemployed, matched employed and overemployed. Overall, respondents reported working 20.0 h per week on average. Differences in reported actual and ideal weekly hours worked across the categories of part-time work were statistically significant. Overemployed respondents reported the highest actual weekly hours worked (23.6) and underemployed respondents reported the highest ideal weekly hours to work (31.0). The hours worked differential (i.e., ideal hours – actual hours) was significantly different across the categories of part-time work. A total of 54.1 % of underemployed respondents reported that their ideal weekly hours to work were more than 30 h (i.e., working full-time). Among overemployed respondents, 14.1 % reported that working zero hours per week (i.e., not working) was ideal. Differences in perceptions of job quality across the three categories of part-time work were statistically significant. Underemployed respondents reported the lowest perceptions of job quality.

**Table 2:** Summary of Reported Weekly Hours Worked, Job Quality, and Work-Life Measures for Respondents Working Part-Time, Overall and by Part-Time Work Categories.

	Category of Part-time Work			
	Underemployed	Matched Employed	Overemployed	Overall
Work Characteristics				
	Mean (STD) Min/Max			
Actual Weekly Hours Worked	19.0 (8.6)* 1/30	19.8 (7.3) 1.5/30	23.6 (5.9) 4/30	20.3 (7.7) 1/30
Ideal Weekly Hours to Work	31.0 (9.5)* 4/60	19.8 (7.3) 1.5/30	15.8 (8.5) 0/28	22.7 (10.4) 0/60
Weekly Hours Worked Difference (Ideal – Actual)	12.0 (8.3)* 1/39	0	−7.7 (6.4) (−30/(−0.5))	2.3 (9.6) (−30/39)
Job Quality^1^	14.9 (4.1)* 6/24	17.7 (3.3) 6/24	16.7 (3.6) 7/24	16.6 (3.9) 6/24
Work Life Measures				
Job Stress^2^	14.5 (3.5)* 5/20	13.7 (3.7) 5/20	15.1 (3.2) 6/20	14.3 (3.58) 5/20
Job Satisfaction^3^	7.8 (2.7)* 3/12	9.1 (2.5) 3/12	7.8 (3.07) 3/12	8.3 (2.78) 3/12
Professional Fulfillment^4^	3.1 (0.98)* 1/5	3.4 (0.91) 1.2/5	2.9 (1.08) 1/5	3.2 (0.99) 1/5
Interprofessional Disengagement^5^	1.9 (0.96)* 1/5	1.7 (0.79) 1/4.7	2.1 (0.99) 1/5	1.9 (0.91) 1/5
Work-Home Conflict^6^	2.5 (0.99)* 1/4	2.2 (0.88) 1/4	2.5 (0.89) 1/4	2.4 (0.94) 1/4
Work Exhaustion^5^	2.5 (1.1)* 1/5	2.2 (0.96) 1/5	2.8 (1.1) 1/5	2.5 (1.1) 1/5

Note: *One way ANOVA, *p* < 0.05.

1 Mean is sum of 6 items on a 4-point scale, 1 = Strongly disagree; 2 = Disagree; 3 = Agree; 4 = Strongly agree.

2 Mean is sum of 5 items rated on a 4-point scale, 1 = Not at all stressful; 2 = Not too stressful; 3 = Somewhat stressful; 4 = Highly stressful.

3 Mean is sum of 3 items rated on a 4-point scale, 1 = Very dissatisfied; 2 = Somewhat dissatisfied; 3 = Somewhat satisfied; 4 = Very satisfied.

4 Mean is the per item mean for 6 items rated on a 5-point scale scored from 1 = not at all true; 2 = Somewhat true; 3 = Moderately true; 4 = Very true; 5 = completely true.

5 Mean is the per item mean for 3 items rated on a 5-point scale from 1 = not at all; 2 = Very little; 3 = Moderately; 4 = A lot; 5 = Totally.

6 Mean is for 1 item rated on a 4-point scale, 1 = Strongly disagree; 2 = Disagree; 3 = Agree; 4 = Strongly agree.

In terms of work-life characteristics, there were statistically significant differences in each work-life characteristic across the three categories of part-time work. Matched employed respondents reported the lowest job stress, work-home conflict, work exhaustion and interprofessional disengagement and the highest job satisfaction and professional fulfillment. Overemployed respondents reported the highest job stress, work exhaustion and interprofessional disengagement and the lowest professional fulfillment. Overall, the proportion of respondents reporting mean item scores for PF that were at or above 4.0 (i.e., feeling professionally fulfilled at least a lot of the time) was 17 %. Overall, the proportion of respondents reporting mean item scores for ID and WE that were at or above 4.0 (i.e., experiencing ID and WE at least at lot of the time) was 2 % and 7.4 %, respectively.

Estimates from the multivariate ordinary least squares regression models for job quality and each of the work-life characteristics are contained in Table 3. Underemployed respondents had significantly lower perceptions of job quality relative to matched employed and overemployed respondents. In terms of work-life characteristics, overemployed respondents reported significantly higher job stress, work-home conflict, work exhaustion and interprofessional disengagement and significantly lower job satisfaction and professional fulfillment relative to matched employed respondents. Underemployed respondents reported significantly lower job satisfaction and significantly higher work-home conflict relative to matched employed respondents. Underemployed respondents were significantly different from overemployed respondents for professional fulfillment and work exhaustion; underemployed respondents were significantly more professionally fulfilled and significantly less exhausted.

**Table 3:** Ordinary Least Squares Regression Results for Job Quality and Work Life Measures.

Variable	Job Quality	Job Stress	Work-Home Conflict	Job Satisfaction	Professional Fulfillment	Work Exhaustion	Interpersonal Disengagement
	Coefficient (B) (SE)						
Constant	**16.5 (0.94)**	**14.6 (0.89)**	**2.2 (0.23)**	**6.3 (0.63)**	**2.7 (0.24)**	**3.0 (0.23)**	**1.9 (0.23)**
**Category of Part- Time Work**							
Overemployed	−0.70 (0.38)	**1.1 (0.38)**	**0.22 (0.10)**	**−0.93 (0.26)**	**−0.29 (0.10)**	**0.41 (0.10)**	**0.27 (0.09)**
Underemployed	**−2.7 (0.35)**	0.46 (0.35)	**0.36 (0.09)**	**−0.79 (0.24)**	−0.06 (0.09)	0.04 (0.09)	0.14 (0.09)
Matched Employment	Ref	Ref	Ref	Ref	Ref	Ref	Ref
**Planned Comparison**							
Underemployed - Overemployed	**−2.0 (0.40)**	−0.65 (0.39)	0.14 (0.10)	0.15 (0.28)	**0.23 (0.10)**	**−0.37 (0.10)**	−0.13 (0.10)
**Setting**							
Independent, Small Chain, Health system retail	**2.9 (0.41)**	**−2.7 (0.38)**	**−0.30 (0.10)**	**2.0 (0.28)**	**0.71 (0.11)**	**−0.89 (0.10)**	**−0.24 (0.10)**
Hospital Inpatient	**2.0 (0.40)**	**−2.6 (0.39)**	**−0.20 (0.10)**	**2.1 (0.27)**	**0.65 (0.10)**	**−0.89 (0.10)**	**−0.27 (0.10)**
Other Patient Care	**3.2 (0.45)**	**−3.2 (0.47)**	**−0.45 (0.11)**	**2.5 (0.31)**	**0.68 (0.12)**	**−0.98 (0.11)**	**−0.34 (0.11)**
Ambulatory Care	**2.8 (0.62)**	**−3.9 (0.60)**	**−0.51 (0.16)**	**2.6 (0.43)**	**0.65 (0.16)**	**−1.1 (0.16)**	−0.13 (0.16)
Large Chain, Mass Merchandiser, Supermarket	Ref	Ref	Ref	Ref	Ref	Ref	Ref
**Marital**							
Single	0.24 (0.60)	0.30 (0.58)	−0.06 (0.15)	−0.61 (0.40)	−0.20 (0.15)	0.25 (0.15)	0.17 (0.14)
Divorced/Widowed/ Separated	0.15 (0.55)	0.02 (0.53)	−0.01 (0.14)	−0.54 (0.37)	−0.16 (0.14)	0.16 (0.14)	−0.08 (0.13)
Married	Ref	Ref	Ref	Ref	Ref	Ref	Ref
Missing	−4.1 (2.5)	1.3 (2.3)				**1.5 (0.54)**	
**Gender**							
Male	0.32 (0.39)	−0.51 (0.37)	−0.11 (0.10)	−0.12 (0.27)	−0.03 (0.10)	**−0.14 (0.10)**	−0.13 (0.10)
Non-binary	−0.72 (2.5)	−0.76 (2.3)	0.16 (0.65)	2.0 (1.8)	0.16 (0.66)	−0.50 (0.66)	−0.44 (0.63)
Female	Ref	Ref	Ref	Ref	Ref	Ref	Ref
**Age**							
24–35	Ref	Ref	Ref	Ref	Ref	Ref	Ref
36–45	**−1.1 (0.51)**	0.39 (0.50)	0.07 (0.13)	−0.51 (0.35)	−0.09 (0.13)	0.17 (0.13)	0.07 (0.13)
46–55	**−1.0 (0.47)**	0.34 (0.46)	−0.04 (0.12)	−0.23 (0.32)	−0.05 (0.12)	0.01 (0.12)	−0.12 (0.12)
56–65	**−1.8 (0.50)**	−0.61 (0.49)	−0.06 (0.12)	−0.38 (0.34)	−0.07 (0.13)	−0.11 (0.13)	−0.06 (0.12)
>65	−0.66 (0.60)	**−1.2 (0.58)**	−0.18 (0.15)	0.79 (0.41)	0.29 (0.16)	**−0.48 (0.15)**	−0.21 (0.15)
**Race**							
White	0.27 (0.71)	1.1 (0.67)	−0.05 (0.17)	**1.3 (0.46)**	0.27 (0.17)	−0.12 (0.17)	−0.11 (0.17)
Asian	0.38 (0.83)	**1.9 (0.78)**	−0.11 (0.20)	0.89 (0.54)	0.20 (0.20)	−0.06 (0.20)	−0.12 (0.20)
Other	0.29 (0.95)	1.4 (0.91)	−0.25 (0.24)	**1.8 (0.64)**	0.36 (0.24)	−0.28 (0.24)	−0.08 (0.23)
Black	Ref	Ref	Ref	Ref	Ref	Ref	Ref
Missing	−1.0 (1.9)	2.3 (1.7)					
**Actual Hours Worked Weekly**	−0.01 (0.02)	0.02 (0.02)	**0.02 (0.01)**	−0.003 (0.01)	−0.01 (0.01)	**0.01 (0.005)**	**0.01 (0.005)**
**Adj. R**^**2**^	0.21	0.22	0.09	0.23	0.14	0.28	0.07

Note: **BOLD**: p < 0.05.

In terms of demographic and work setting characteristics, respondents in each work setting category reported significantly higher perceptions of job quality relative to respondents working in large chain, mass merchandiser and supermarket settings. Respondents 36–65 years of age had significantly lower perceptions of job quality relative to respondents 35 years and younger. Asian respondents reported significantly higher job stress relative to black respondents. White respondents and respondents in the Other race category reported significantly higher job satisfaction relative to black respondents. Generally, respondents working in large chain, mass merchandiser and supermarket practice settings reported significantly lower job satisfaction and professional fulfillment and significantly higher job stress, work-home conflict, work exhaustion and interprofessional disengagement relative to respondents working in each of the other practice settings.

## Discussion

This is the first known study to classify pharmacists working part-time as underemployed, overemployed, or matched employed and to associate the mismatch between actual and desired hours worked with work-life characteristics. The results show that for pharmacists working part-time, a lack of control over how much they work is negatively associated with job quality and work-life characteristics relative to pharmacists with work schedule control.

Approximately one-third of respondents working part-time were underemployed. Of respondents classified as underemployed, over one-half (54.1 %) wanted to work full time, suggesting that the unavailability of full-time employment was not ideal. According to labor economic theory, one explanation for underemployment of pharmacists is the surplus of pharmacists, or the surplus of hours pharmacists were supplying to the labor market where the respondents were working, which created a loose, local labor market. Consequently, there likely was a small number of unfilled full-time jobs available or a small number of unfilled part-time hours available to offer pharmacists.^34^, ^35^, ^36^, ^37^, ^38^, ^39^ Alternatively, full-time employment opportunities could have been limited if part-time employment was less expensive for employers, due to not having to offer health insurance and other costly benefits associated with full-time employment.^34^, ^35^, ^36^, ^37^, ^38^, ^39^ The explanations for underemployment may provide an explanation for the reduction in the proportion of pharmacists working part-time in 2019 relative to previous years.

The results showed that approximately one-quarter (23.8 %) of respondents working part- time were categorized as overemployed. One explanation for being overemployed is that there may have been a shortage of pharmacists, or a shortage of hours pharmacists were supplying to the labor market where the overemployed respondents were working. Respondents likely had the option of accepting or not accepting the additional work hours that were offered by their employers.^40^^,^^41^ The 2019 NPWS did not assess whether respondents refused to work additional hours that were available to them. Future research could explore local pharmacist labor market variables such as characteristics of jobs, employer hiring practices and adequacy of pharmacist supply to explain overemployment, underemployment and matched employment of pharmacists.

By comparing data reported in the 2019 NPWS about ideal and actual hours worked weekly, this study produced a potentially more accurate estimate of the proportion of respondents working part-time who were underemployed, relative to the BLS definition of underemployment of pharmacists working part-time.^42^ It is unknown whether the prevalence of underemployment, overemployment and matched employment among pharmacists working part-time was greater in 2019 relative to previous years. Future research could begin to measure underemployment, overemployment and matched employment of pharmacists working part-time, adopting the approach used in this study. Additionally, future research could examine reasons why pharmacists working part-time are overemployed or underemployed. This information will be particularly useful given projections of a pharmacist shortage now and into the future, which could reduce the likelihood of underemployment and increase the likelihood of overemployment as pharmacist labor markets become less tight (i.e., fewer pharmacists to work needed hours).^12^^,^^40^^,^^41^ Tracking trends in the prevalence of overemployment and underemployment could be an additional method to assess the tightness of the pharmacist labor market locally, regionally and nationally.

The significant differences between overemployment and matched employment for all six of the work-life characteristics and for two of the six work-life characteristics between underemployment and matched employment suggest the benefit to part-time pharmacists of having work schedule control (i.e., matched employment).^29^^,^^33^ One explanation for the non- significant difference between underemployment and matched employment for job stress, professional fulfillment, work exhaustion and interpersonal disengagement is that underemployed respondents were not exposed to work environments (i.e., worked fewer hours) as much as matched respondents. Thus, their negative perceptions of these characteristics did not outweigh the benefits of matched employment. An important topic for future research is learning more about matched employment by pharmacists working part-time. For example, what strategies do pharmacists use to avoid working additional hours that they do not desire to work? What age-related or life-cycle characteristics, such as perceptions of income adequacy, are associated with matched employment? Do matched employed part-time pharmacists have better physical, mental and emotional health compared to overemployed and underemployed pharmacists?^29^^,^^33^

The results also suggest that poor work schedule control appears to be no worse for pharmacists working part-time who are overemployed versus underemployed as evidenced by no significant differences between overemployment and underemployment for four of the six work-life characteristics. How poor work schedule control manifests in overemployment and underemployment could be a topic for future research. According to the Conservation of Resources Theory, poor work schedule control can disrupt planning for time to be spent in enjoyable leisure activities and household activities such as attending children's events or childcare responsibilities, respectively.^56^^,^^57^ The stress resulting from disrupted leisure and household activities may carry over to work, increasing job stress and work exhaustion and reducing enjoyment and fulfillment from work.^56^^,^^57^ Additional family characteristics such as family income level, perceived adequacy of family income and number and age of children, as well as including the reasons why pharmacists want to either work more (i.e., professional esteem goals, more family income) or fewer hours (i.e., spend more time with family, reduce childcare costs) could be included in future research.^56^^,^^57^ Perceptions of inadequate income leading to financial stress, especially for young pharmacists who are underemployed and have student loan debt, could also be included.^58^^,^^59^ Additionally, future research could examine the strategies respondents adopt to deal with the stress of underemployment and overemployment and the impact of the strategies on psychosocial aspects of work and mental (i.e., depression, anxiety, psychological stress), physical (i.e., physical pain and distress) and emotional (i.e., stress about finances) health.^22^^,^^29^

### Limitations

Although the response rate to the survey was 4.9 %, which is reasonable for an electronically administered survey, analysis of non-response bias to the survey showed that proportionally more responses were received from female pharmacists and older pharmacists, relative to population characteristics.^49^ Since the results of the present study showed a higher proportion of females and older respondents working part-time, the results for the point estimate of the proportion of actively practicing pharmacists who are working part-time may be biased upward. Future research could explore methods to weight the respondent sample to population estimates to refine the point estimate.

The data represent perceptions of jobs and work life at a single “snapshot’ in time. As such, no causative statements can be made about underemployment and overemployment for actively practicing pharmacists. Future research could employ longitudinal approaches to explore how movement in and out of overemployment and underemployment impact decision making and work life characteristics and pharmacist health.^22^^,^^29^

A respondent's work setting was significantly associated with a respondent being categorized as underemployed, matched employed or overemployed and with the work life characteristics. Whether underemployment or overemployment across work settings is associated with the economic situation of the pharmacies, characteristics of local labor markets, organizational-specific hiring/employment practices, or preferences and characteristics of respondents beyond characteristics controlled for in the multivariate models is unknown and could be topics for future research.

## Conclusion

The higher rate of underemployment among pharmacists working part-time is consistent with the surplus of US pharmacists in 2019. Overemployed and underemployed respondents (i.e., those with poor work schedule control) reported significantly lower levels of several of the work life outcomes relative to respondents with matched employment. One area for future research is examining how pharmacists' own health and their families are impacted by decisions to work-part-time and work more or fewer hours than is ideal using relevant theoretical frameworks. Improving pharmacists' well-being and sustaining the pharmacist workforce are important given the projected shortage of US pharmacists.

## CRediT authorship contribution statement

**David A. Mott:** Writing – review & editing, Writing – original draft, Validation, Supervision, Methodology, Investigation, Formal analysis, Data curation, Conceptualization. **William R. Doucette:** Writing – review & editing, Validation, Supervision, Resources, Project administration, Methodology, Investigation, Funding acquisition, Data curation, Conceptualization. **Eilan Alhersh:** Writing – original draft, Investigation, Formal analysis, Conceptualization. **Vibhuti Arya:** Writing – review & editing, Investigation, Conceptualization. **Brianne K. Bakken:** Writing – review & editing, Investigation, Conceptualization. **Caroline Gaither:** Writing – review & editing, Methodology, Investigation, Conceptualization. **David H. Kreling:** Writing – review & editing, Methodology, Investigation, Conceptualization. **Jon C. Schommer:** Writing – review & editing, Methodology, Investigation, Conceptualization. **Matthew Witry:** Writing – review & editing, Resources, Methodology, Investigation, Funding acquisition, Data curation, Conceptualization.

## Funding

This project was funded by the Pharmacy Workforce Center, Inc.

## Declaration of competing interest

The authors declare the following financial interests/personal relationships which may be considered as potential competing interests:

David Mott reports financial support was provided by Pharmacy Workforce Center, Inc. If there are other authors, they declare that they have no known competing financial interests or personal relationships that could have appeared to influence the work reported in this paper.
